# Fracture of the Penis

**Published:** 1897-10

**Authors:** 


### FRACTURE OF THE PENIS.

A man in a very drunken state had a powerful erection, and without any cause or reason known to himself he with both hands bent it forcibly backward. He was conscious of something giving away, and awakened with the pain. The erection subsided after a time, and the pain also, but he was very much surprised in the morning by the enormous dimensions the organ had assumed, and its color. He therefore hastened to the hospital. With the exception of the glans, the organ was swollen and of a dark violet color. On the dorsum was a painful thickening, when the surgeon suspected a rupture of the corpus cavernosum had taken place. The treatment consisted of rest in bed, cold compresses and lead lotion, and in fourteen days the normal color had returned, although the thickening remained almost unchanged. In six weeks the patient showed that, although a slight thickening still remained, no lasting injury had been sustained.—*Deutsche Med. Zeit.—N. E. Medical Monthly.*

